# Quantitative mapping of DNA phosphorothioatome reveals phosphorothioate heterogeneity of low modification frequency

**DOI:** 10.1371/journal.pgen.1008026

**Published:** 2019-04-01

**Authors:** Jinli Li, Yi Chen, Tao Zheng, Lingxin Kong, Sucheng Zhu, Yihua Sun, Zixin Deng, Litao Yang, Delin You

**Affiliations:** State Key Laboratory of Microbial Metabolism, Joint International Research Laboratory of Metabolic and Developmental Sciences, and School of Life Sciences & Biotechnology, Shanghai Jiao Tong University, Shanghai, China; UNITED KINGDOM

## Abstract

Phosphorothioate (PT) modifications of the DNA backbone, widespread in prokaryotes, are first identified in bacterial enteropathogens *Escherichia coli* B7A more than a decade ago. However, methods for high resolution mapping of PT modification level are still lacking. Here, we developed the PT-IC-seq technique, based on iodine-induced selective cleavage at PT sites and high-throughput next generation sequencing, as a mean to quantitatively characterizing the genomic landscape of PT modifications. Using PT-IC-seq we foud that most PT sites are partially modified at a lower PT frequency (< 5%) in *E*. *coli* B7A and *Salmonella enterica* serovar Cerro 87, and both show a heterogeneity pattern of PT modification similar to those of the typical methylation modification. Combining the iodine-induced cleavage and absolute quantification by droplet digital PCR, we developed the PT-IC-ddPCR technique to further measure the PT modification level. Consistent with the PT-IC-seq measurements, PT-IC-ddPCR analysis confirmed the lower PT frequency in *E*. *coli* B7A. Our study has demonstrated the heterogeneity of PT modification in the bacterial population and we also established general tools for rigorous mapping and characterization of PT modification events at whole genome level. We describe to our knowledge the first genome-wide quantitative characterization of PT landscape and provides appropriate strategies for further functional studies of PT modification.

## Author summary

Phosphorothioate (PT) modification is a novel DNA modification, previous studies showed that PT modifications in *E*. *coli* occure at G_ps_AAC/G_ps_TTC motifs, but the modification frequency at each site are not known. In this study, we introduced two methods: PT-IC-seq, which could quantitatively characterize the genomic landscape of PT modifications; and PT-IC-ddPCR, which could measure PT modification frequency precisely. Through these two new methods, heterogeneity, an important feature of PT modification, is revealed intuitively and accurately. The modifications revealed in this study provide a basis for the final revelation of the physiological functions of PT and give valuable reference for other DNA modification studies.

## Introduction

Phosphorothioate (PT) modification of DNA, in which the non-bridging oxygen in the phosphate moiety of the sugar-phosphate backbone is replaced by sulfur, is widespread in prokaryotes in and *R*-configuration and a sequence-selective manner. PT modification was originally developed as an artificial means to stabilize oligodeoxynucleotides against nuclease degradation[1]. A physiological PT modification from bacterial enteropathogens *Escherichia coli* B7A was first identified by liquid chromatography-coupled tandem quadrupole mass spectrometry (LC-MS/MS) analysis in 2007[2]. The physiological PT modifications are governed by a large family of five-gene clusters termed as *dndA-E*[3, 4]. The biochemical study of PT modification revealed that PT-modifying enzymes DndACDE function as a large protein complex, with DndB actings as a negative transcriptional regulator[5–7]. Many bacterial PT-modifying enzymes act in concert with and are encoded in close proximity to cognate restriction endonucleases DndFGH. PT modifications catalysed by PT-modifying enzyme protects DNA from digestion by DndFGH with which it forms a restriction-modification (R-M) system[8]. The novel R-M systems protect cells from invading foreign DNA similar to methylation-based R-M systems[9]. However, more than half of all PT bacterial strains lack the *dndF-H* restriction system in spite of containing *dndA-E* and PT. The fact that many strains of bacteria lack the restriction enzyme components of a typical R-M system is consistent with the idea that PT modifications and *dndA-E* genes provide functions other than R-M, such as control of gene expression[10].

PT modifications occur in DNA in a sequence-specific manner, the LC-MS/MS method was first applied to define the PT sequence contexts and quantify PT modifications in bacteria, and all 16 possible PT-linked dinucleotides in *R*_P_ configuration have been detected. In *E*. *coli* B7A, PT modifications occur in G_ps_A and G_ps_T (phosphorothioate-containing dinucleotides) at 370 ± 11 and 398 ± 17 PTs per 10^6^ nt nucleotides, respectively[11]. LC-MS/MS method utilizes the PT-induced nuclease inhibition that produces a limit digest of PT-containing dinucleotides, and thus is limited to quantitative analyses of dinucleotides such as d(G_ps_A) and d(G_ps_T) but not genomic mapping of PT modifications, termed phosphorothioatome. Recently, two sequencing technologies were applied to map bacterial phosphorothioatome: single-molecule, real-time (SMRT) sequencing and deep sequencing of iodine-induced cleavage at PT (ICDS)[10]. The double-stranded PT modification in *E*. *coli* B7A was determined by both SMRT and ICDS to occur in the G_ps_AAC and G_ps_TTC sequence context, which is consistent with the previous observation of equimolar quantities of d(G_ps_A) and d(G_ps_T) detected in the genome by LC-MS/MS. Only 7045 ± 470 out of 40701 possible GAAC/GTTC sites in B7A genome were detected as PT-modified in spite of the presence of the PT-dependent R-M system in this strain, indicating that the genome-wide PT distribution is partial in the B7A genome[10]. Similar partial modification has been detected in DNA methylome, which is also been characterized as epigenetic heterogeneity[12, 13]. DNA methylation heterogeneity is widespread in organisms, which play critical roles in cell physiology, including as a source of phase variation that increase population-level phenotypic plasticity and provides opportunities to modulate transcription in response to changing environmental conditions, the colonization of animals by bacterial pathogens[14–16], and cellular development and tumorigenesis[17, 18]. Here, we developed two techniques that provide improved resolution to observe heterogeneity of PT modifications: an iodine induced cleavage-based PT sequencing (PT-IC-seq) and an iodine induced cleavage-based PT droplet digital PCR (PT-IC-ddPCR). Our data reveal PT heterogeneity in bacteria. These characterizations of PT modification will open the door to understanding biology of this novel DNA modification.

## Results

### Discovery of new PT sites in bacteria genome using deep resequencing

**Fig 1:**
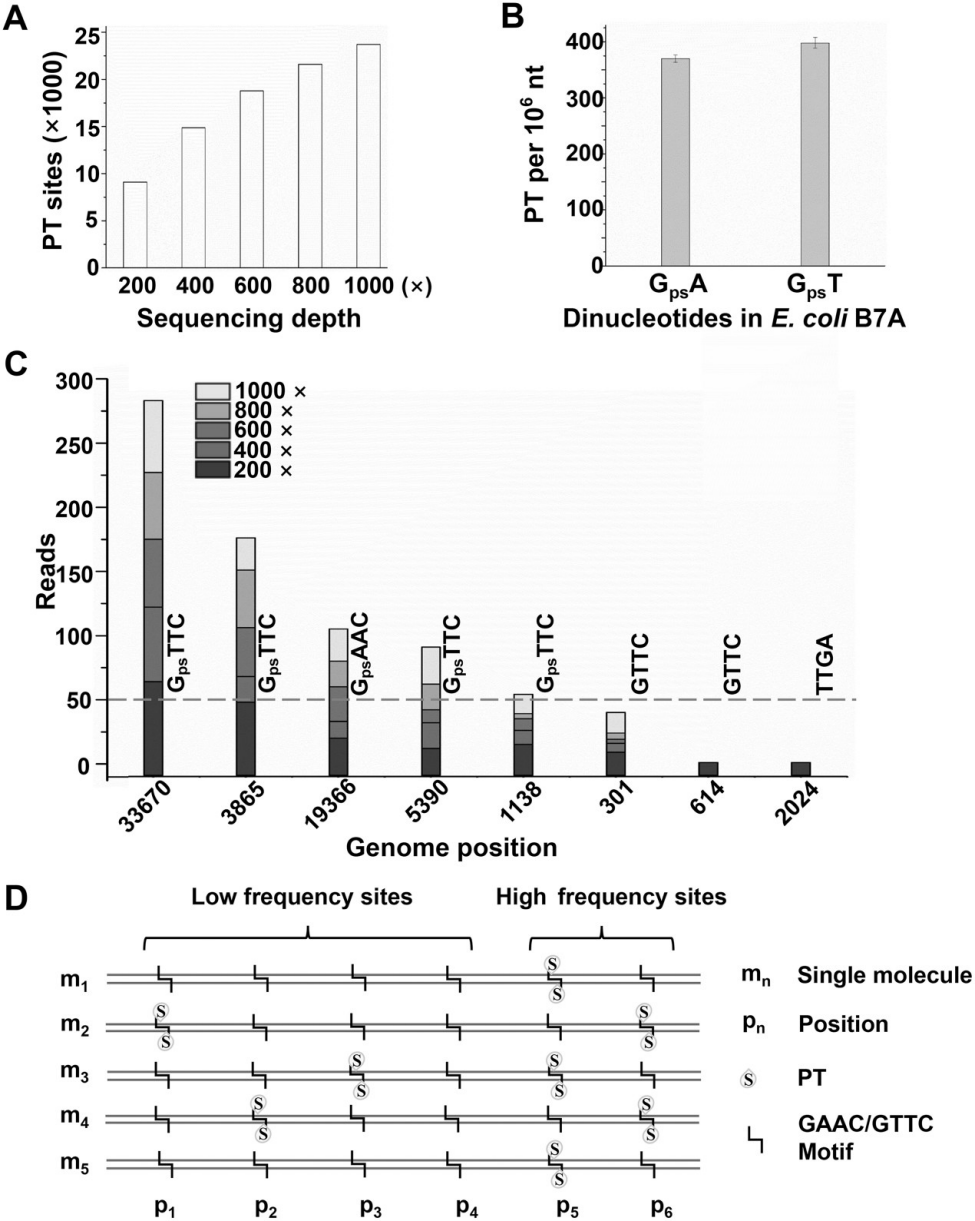
Discovery of new PT sites using deep resequencing and the inconsistency between extensive PT modified sites and the PT linked dinucleotides abundance of the *E*. *coli* B7A genome. (A) Plotted PT modified sites detected with the increase of sequencing depth. (B) The PT abundances quantified by LC-MS/MS. Error bars are calculated as the s. d. of three biological replicates. (C) Accumulation of sequencing depth contributed to the revealing of more PT-modified sites. Here, one loci are chosen for each case as an example to illustrate each situation: some sites like loci 33670, the ended reads number of which reached 50 at 200 × was detected as PT modified sites, while some sites like 3865 loci only be regarded as PT modified site when the sequencing depth increase up to 400 ×. In other word, if the sequencing depth is only 200 ×, they would be identified as non-PT modified sites. Similar phenome can be seen at the sites like 19366 loci (600 ×), 5390 loci (800 ×) and 1138 loci (1000 ×). Also, some sites like 301 loci, the ended reads number of which could not reach 50 even the sequencing depth increased up to 1000 ×. While, some GAAC/GTTC sites like 614 loci and some randomly selected non GAAC/GTTC sites like 2024 loci, the ended reads number could not be detected at each five gradient sequence depth. (D) The schematic molecular PT modification. The specific GAAC/GTTC site is partially modified and with different modification frequency. High frequency sites are easy to detected and low frequency sites need further sequencing depth.

DNA sequencing is an essential tool for biological and medical studies, and it can be used not only to determine the precise order of the four nucleotide bases in genetics, but also to analyze specific nucleotide base in epigenetics. Recently, we developed ICDS approach [10] to map PT locations in bacterial genomes based on adaptation of high-throughput next generation sequencing (NGS) technology. Using this approach, only 7045 ± 470 out of 40,701 total possible PT modified sites across the *E*. *coli* B7A genome were detected to be PT modified sites[10]. Mapping the detected modification sites in the *E*. *coli* B7A genome showed that G_ps_AAC/G_ps_TTC distributs sporadically throughout the whole genome. Deep resequencing has been recently used for the correction of genome sequence[19], rare genetic variation[20, 21], and discovery of large numbers of single nucleotide polymorphisms (SNPs)[22]. For constructing more accurate high-resolution genetic map of PT sites in the *E*. *coli* B7A, we conducted resequencing of *E*. *coli* B7A genome DNA by using ICDS approach with increasing sequencing depth. The genomic DNA from *E*. *coli* B7A was isolated and treated with iodine (S1 Fig). When sequencing depth was increased to approximately 1000 ×, 23,708 out of a total of 40,701 GAAC/GTTC sites were detected as PT modification sites (Fig 1A and S1 Table). The newly detected PT sites were also distributed sporadically throughout the *E*. *coli* B7A genome. The number of PT sites were ~3 times more than the 7,045 sites previously detected by low coverage sequencing (200×). If all of these 23,708 GAAC/GTTC sites are fully modified homogenously in every cell, it would corresponds to ~4,800 PTs per 10^6^ nt in G_ps_A or G_ps_T motif, which was ~12 times more than the ~384 PTs per 10^6^ nt quantified by LC-MS/MS previously[11]. This suggests that there are significant heterogeneity in the PT modifications, where the individual GAAC/GTTC sites were phosphotrionated in some cells but not all. The detection of a larger number of PT modification sites is likely due to the increased sequencing depth, which led to higher sensitivity in the detection of GAAC/GTTC sites with low frequency PT modification.

In order to confirm the above finding, we firstly re-quantified PTs in *E*. *coli* B7A by LC-MS/MS to rule out the effects of biological samples. Consistent with the previous measurements, PT modifications at the same level occurred in G_ps_A and G_ps_T contexts at 370 ± 11 and 398 ± 17 PTs per 10^6^ nt, respectively (Fig 1B). We then randomly sampled the sequencing data to analyze the involvement of the detected PT sites in different sequencing depths (200, 400, 600, 800 and 1000 × coverage). The number of G_ps_AAC/G_ps_TTC sites achieved were plotted in Fig 1A. 9,098, 14,867, 18,770, 21,596, 23,708 GAAC/GTTC sites across the *E*. *coli* B7A genome were detected as PT modified sites at 200, 400, 600, 800 and 1000 × coverage, respectively. As expected, with the increase of sequencing depth, the number of PTs detected increased rapidly. To further verify the sequencing depth required for the detection of PT sites at low-frequency, we subsampled the sequencing data. The analysis of reads correlation showed most relatively low-reads (< 50) sites (low-frequency sites) could be identified as modification sites, since their reads increased with the increase of sequencing depth (Fig 1C and S2 Table). In contrast, the reads from some non-phosphorothioated GAAC/GTTC sites and randomly selected ten other sequence motifs such as CCGA, TTGA, ACGG, were not increased with the increase of sequencing depth (Fig 1C and S3 Table). These results suggested that most GAAC/GTTC sites on *E*. *coli* B7A genome could be modified at a lower frequency (Fig 1D).

### Development of iodine cleavage and next generation sequencing for quantitatively PT mapping

**Fig 2:**
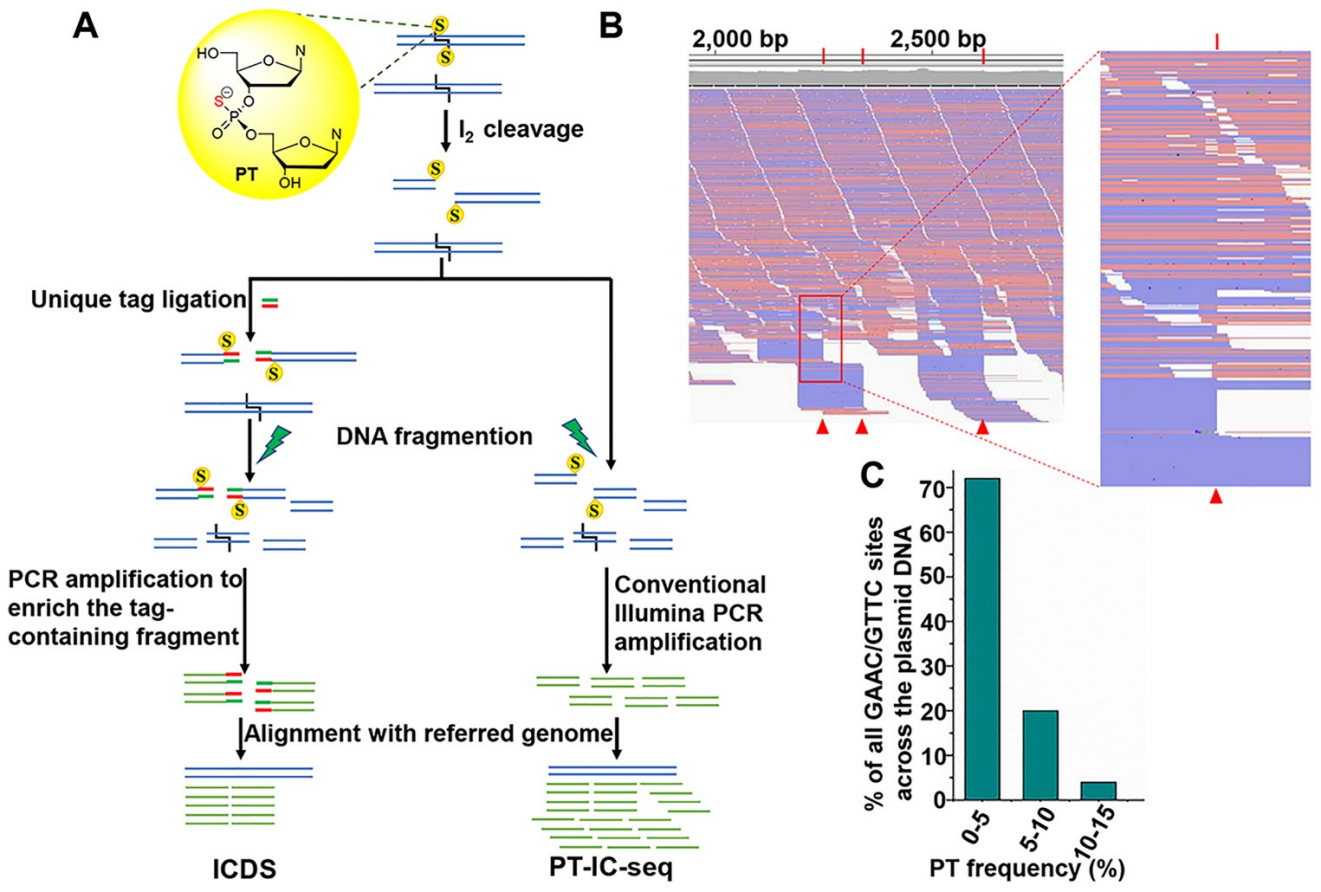
PT-IC-seq, a new method for quantitatively PT mapping. (A) Flowchart of ICDS and PT-IC-seq. The treatment of bistranded PT modified DNA with I_2_ will produce a double-strand break. In the ICDS method, after treatment with I_2_, the resulted double-strand break is then processed and ligated to a unique double-stranded oligodeoxynucleotide tag, followed sonicated to an optimal range (150–350 bp) in length. After Illumina adaptors addition, the DNA is subjected to PCR amplification to enrich the I_2_ cleaved fragments. And finally, subjected to Illumina sequencing. While, in the PT-IC-seq method, after treatment with I_2_, the resulted double-strand broke into long DNA fragments were directly fragmented. Following addition of Illumina adaptors, the DNA is subsequently subjected to PCR amplification and sequencing. (B) The snapshot of genome browser (IGV) representing a partially PT modified sites in pBlueScript SK(+) plasmid extract from *S*. *enterica* serovar Cerro 87. The top track shows counts of 5’ ends in the selected region. Blue segments represent reads mapped to the minus strand and red segments represent reads mapped to the plus strand. The red triangle marks G_ps_AAC/G_ps_TTC site. (C) Statistics analysis of the PT modification frequency in pBlueScript SK(+) plasmid extract from *S*. *enterica* serovar Cerro 87. All of the PT modified sites in the pBlueScript SK(+) plasmid extract from *S*. *enterica* serovar Cerro 87 are partially-PT modified, and more than 70% of the PT modified sites with the PT ratio lower than 5%.

To investigate PT modification frequency in genome landscape at single-base resolution, we sought to develop a high-throughput assay to quantitatively determine the PT modification percentage at each site. In the ICDS approach, iodine reagent was introduced to cleave DNA at G_ps_AAC/G_ps_TTC sites, and then ligated to an adaptor with specific index sequence for enriching the DNA fragments with PT modifications by PCR amplification. Since the PCR amplification step will result in enriched amplicons with PT modifications, ICDS sequencing approach can only be used for identifying PT sites but not for quantification. Based on these considerations, we developed one approach named iodine induced cleavage-based PT sequencing (PT-IC-seq) to quantitatively determine the PT modification percentage without enrichment process. As shown in Fig 2A, DNA sample was treated with iodine and then sonicated to 150–350 base pair fragments, end-repaired, adenylated, and ligated to Illumina adapters. After PCR amplification, a standard Illumina DNA library was constructed for high-throughput sequencing. The modified G_ps_AAC/G_ps_TTC motifs would be cleaved and should be presented as the reads ends in the sequencing output. The unmodified GAAC/GTTC motifs would not be cleaved and should be present in the internal locations of DNA fragments. The ratio of a specific GAAC or GTTC sites with sequence reads at the end versus internal represents the relative PT modified to unmodified ratio. Through mapping sequencing reads to the reference genome, PT modification frequency for every GAAC or GTTC site can be calculated.

To test the feasibility of PT-IC-seq for determining PT modification frequency, the DNA of plasmid BlueScript SK(+) from *Salmonella enterica* serovar Cerro 87 we isolated and tested as an example. By aligning the output reads to the plasmid sequence, as expected, we found that the most of intact GAAC or GTTC motifs appeared at the internal of sequencing reads, which should be from unmodified GAAC/GTTC sites, and some of sequencing reads were initiated with AAC or TTC, which resulted from the double-strand cleavage of PT modified G_ps_AAC/G_ps_TTC by iodine (Fig 2B). For evaluating the PT level of individual GAAC/GTTC sites, we counted and calculated the ratio of reads obtained from fragment terminals to total reads of each site. The PT modification level of all 25 GAAC/GTTC sites throughout the entire plasmid were calculated and shown in S4 Table. Meanwhile, to exclude the false-ended reads generated by random shearing, eight non GAAC/GTTC sites were randomly selected as control sites. The false PT detection possibility was estimated to be below 1% (~0.54%, S4 Table). Statistics analysis of PT modification frequency showed that most of the GAAC/GTTC sites (72%) were modified with a low PT modification percentage of < 5% (Fig 2C and S4 Table). These results demonstrated that the PT-IC-seq approach could quantitatively determine the PT ratio of each GAAC/GTTC site and was suitable for investigating PT modification frequency of whole genome simultaneously.

### PT-IC-seq of the *E*. *coli* B7A genome reveals PT modification at low frequency

**Fig 3:**
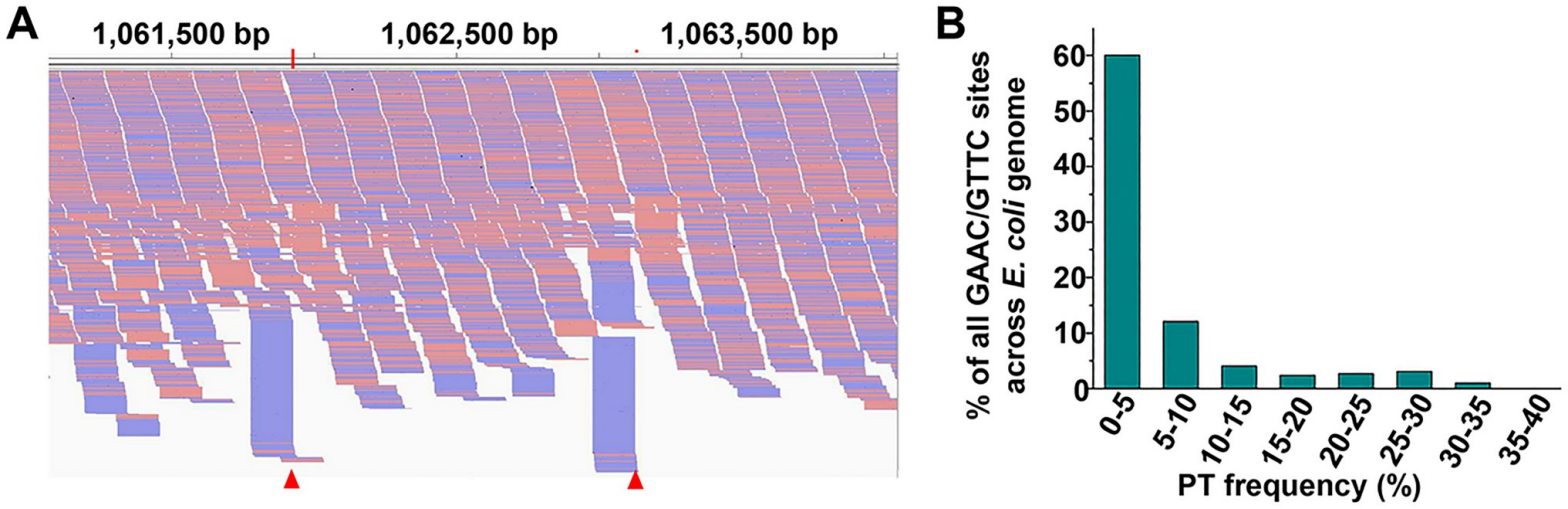
PT-IC-seq of *E*. *coli* B7A genome reveals low PT modification frequency globally. (A) The snapshot of genome browser representing partially modification site in bacteria genome. The top track shows counts of 5’ ends in the selected region. Blue segments represent reads mapped to minus strand and red segments represent reads mapped to plus strand. The red triangle marks two partial PT sites in *E*. *coli* B7A genome (B) The ratio of the I_2_-specific-cleavaged reads to all the reads across the site and the PT ratio falling in the range of proportions. Statistics analysis of the PT modification frequency in the genomes of *E*. *coli* B7A. All of the PT modified sites in the genomes of *E*. *coli* B7A are partially-PT modified, and about 60% of the PT modified sites with the PT ratio lower than 5%.

Given the successful detection of PT modification percentage of each modified sites in plasmid DNA, the PT-IC-seq approach was then applied to genomic DNA isolated from *E*. *coli* B7A. The obtained sequencing reads were mapped to a reference genome of *E*. *coli* B7A (GenBank accession No. CP005998.1). About 85 percent of all GAAC/GTTC sites (34,796/40,701) across the *E*. *coli* B7A genome were detected with PT modification, and the PT modification frequency of each site was shown in S5 Table. The false PT detection possibility was estimated to be below 1% (~0.62%, S5 Table). Among the 34,796 PT modification GAAC/GTTC sites, 24,477 sites (60.1%) were found to have a modification percentage below 5% (Fig 3B), which was consistent with the results of plasmid that 72% of the total detected PT sites were modified with a low PT ratio. The number of PT sites calculated by PT-IC-seq is 392.4 per 10^6^ bp, which was very consistent with the MS measurements of 370 ± 11 G_ps_A and 398 ± 17 G_ps_T per 10^6^ nt. In addition, 10,326 5,379, 2,718 and 360 GAAC/GTTC sites were identified with higher PT frequencies than 5%, 10%, 20% and 30%, respectively. Only two sites’ PT ratio exceed 35% were 35.36% and 35.17% respectively. The low modifcation frequency suggests heterogeneity of PT modification in the bacterial population. That is, only a small proportion of individual cells contain PT at specific sites, whereas other cells do not. The low frequency PT modification may indicate distinctive roles yet to be identified. The majority of G_ps_AAC/G_ps_TTC sites were modified with a low percentage was also consistent with the phenomenon that a large part of GAAC/GTTC motif could be PT modified while the actually detected G_ps_A and G_ps_T are only at low level (< 400 PTs per 10^6^ nt).

By comparing the 360 high-frequency sites (>30%) and the 9098 PT sites determined by the ICDS approach with 200 × coverage, we found that 353 out of the 360 (98.06%) sites with a modification frequency more than 30% overlap with the 9098 PT modified sites detected by ICDS with 200 × coverage(S6 Table). Meanwhile, 8391 out of the 9098 (92.2%) PT modified sites detected by ICDS with 200 × overlap with the 10319 sites with a modification frequency more than 5% by PT-IC-seq. The result from these comparisons shows that the sites with higher modification frequency detected by PT-IC-seq can also be detected as PT modified sites by ICDS with lower sequencing depth. These results indicate that the two sequencing methods are very consistent for detecting PT modification sites.

**Fig 4:**
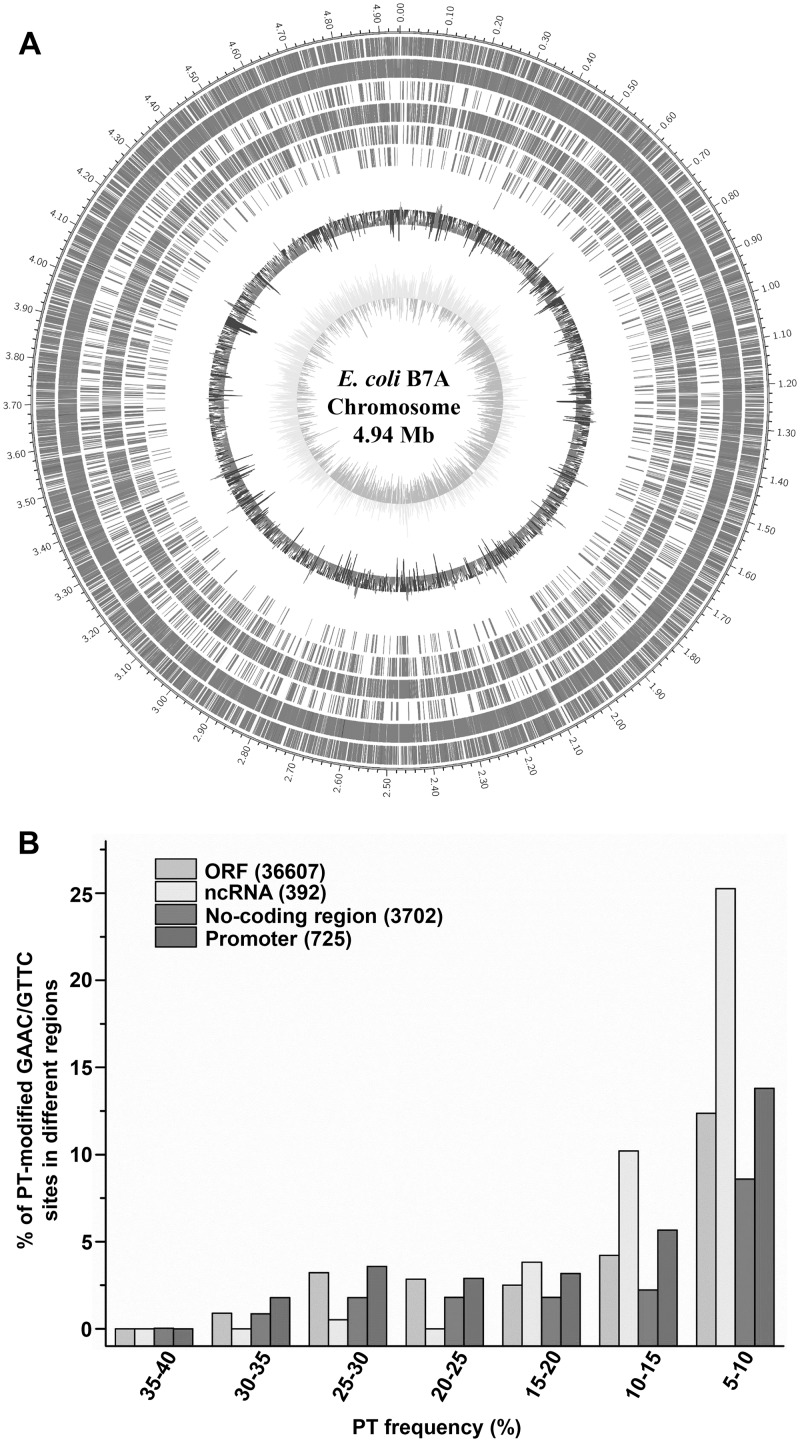
The distribution analysis of PT sites with different modification frequency. (A) PT site mapping across the *E*. *coli* B7A genome. From outer to inner circles: 1 to 7 (PT sites modified with 5%-10%, 10%-15%, 15%-20%, 20%-25%, 25%-30%, 30%-35%, 35%-40%): PT sites in ORFs (grey), in rRNA(yellow), tRNA (blue) and non-coding regions (red); 8: GC content; 9: GC skew. (B) The distribution analysis of PT modification frequency for GAAC/GTTC sites at different region in the genome.

The percentage of PT-modified GAAC/GTTC sites with PT modification frequency > 5% in different regions of the genome varied from 17.1 to 39.8%, with 9,537 of 36,607 sites (26.0%) modified with PT in ORFs, 156 of 392 (39.8%) in ncRNAs and 633 of 3702 (17.1%) in the noncoding regions. Among the non-coding region, there are 224 of 725 sites (30.9%) in the promoter regions modified with PT modification frequency > 5%. The relatively high-frequency (> 20%) PT sites were distributed in ORF and non-coding regions other than ncRNA; while those PT frequency < 20% distributed relatively evenly across the B7A chromosome (Fig 4). We performed an extended motif search based on the 360 high-frequency sites to examine whether there is any additional preference of nucleotides flanking the GAAC or GTTC sequence. However, no additional consensus nucleotides were observed (S7 Table).

### Validation of PT modification heterogeneity by PT-IC-ddPCR

**Fig 5:**
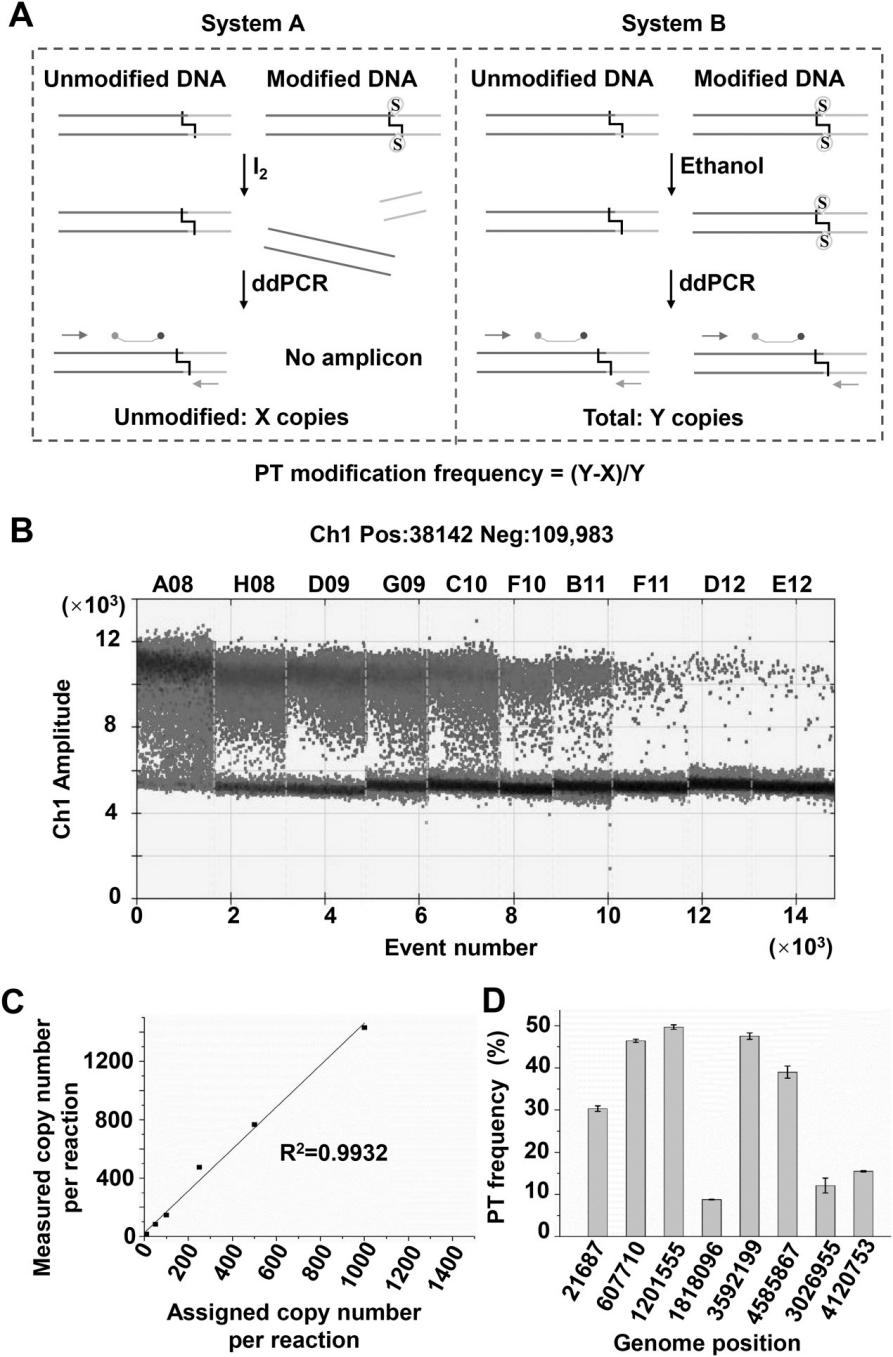
PT-IC-ddPCR quantify PT modification frequency. (A) Schematic diagram of the PT-IC-ddPCR quantified the DNA PT modification frequency at a specific site. Left (System A): DNA sample was firstly treated with iodine, then unmodified DNA was quantified as X copies by ddPCR using the primers and probe; Right (System B): Total DNA was quantified as Y copies by droplet digital PCR using the same primers and probe; the modified DNA was calculated as Y-X and the PT modification frequency was calculated as (Y-X)/Y. (B) The one-dimensional (1D) scatter plot of serial concentration of *E*. *coli* B7A by droplet digital PCR. (C) Dynamic range and correlation of the ddPCR assay for quantification of PT modification frequency at 1818096 loci in *E*. *coli* B7A genome. The observed positive droplets matched well with the predicted values according to Poisson statistics (R^2^ = 0.9932). (D) The PT modification frequency of 8 loci (G1-G8), each value was the mean based on at least three measurements.

In order to evaluate the creditability of the PT-IC-seq quantitative assays, we then developed another method named PT-IC-ddPCR for validation of the PT modification frequency. The PT-IC-ddPCR approach was developed based on iodine-induced selectively cleavage at PT sites and inherent property of droplet digital PCR (ddPCR) technique for absolute DNA quantification. In the PT-IC-ddPCR (Fig 5A), a portion of the PT modified genome DNA sample (system A) was treated first by iodine. The double strands of those PT modified molecules broke at specific site while the non-modified DNA remained intact. When the designed primers and probes (S8 Table) were used for quantitative ddPCR, only the non-modified DNA was amplified and quantified, and the results were recorded as X copies (Fig 5A, system A). Similary, another equal portion of the DNA sample (system B) was treated by ethanol instead of iodine, the treated DNAs were then quantified by ddPCR, and the obtained results were determined as Y copies (Fig 5A, system B). The Y copies in the system B was the sum of modified and unmodified molecular number at target sites, which theoretically was equivalent to the total DNA number in system A. The number of PT modified molecules at this site was calculated as Y-X. The PT modification frequency of target site can be calculated by the percentage of PT modified molecular to the total sum of the PT modified and non-modified molecular number, which was (Y-X) / Y.

Using the PT-IC-ddPCR method, we first evaluated its dynamic range and correlation employing 1818096 loci in *E*. *coli* B7A genome as an example. The observed positive droplets matched well with the predicted values, and the R^2^ value of 0.9932 indicated that this standard curve had good linearity and the quantified results were accurate with low RSD (all < 25%) (Fig 5B and 5C). The PT modification frequency at this position obtained from PT-IC-ddPCR method is 8.78%, which was consistent with 5.19% obtained from PT-IC-Seq method (S9 Table). PT-IC-ddPCR method were then applied to another 7 sites in *E*. *coli* B7A genome, which were detected as PT modification sites by PT-IC-seq. As can be seen from S9 Table, the PT modification frequency obtained from PT-IC-ddPCR at all 8 sites were consistent with the value obtained from PT-IC-seq method. Therefore, this assay provided locus-specific validation of the PT-IC-seq results. Although the PT ratio value from PT-IC-Seq method is a slightly different from those of PT-IC-ddPCR analysis, the overall trend is highly consistent (S9 Table). As is shown in Fig 5D, all the 8 sites’ PT modification frequency in a population of DNA molecules were no more than 50%. The PT-IC-ddPCR assay identified 8 GAAC/GTTC sites were partially modified and demonstrated PT modification has heterogeneity in *E*. *coli* B7A genome.

### Quantitative mapping of PT sites in the *Salmonella* genome using PT-IC-seq

**Fig 6:**
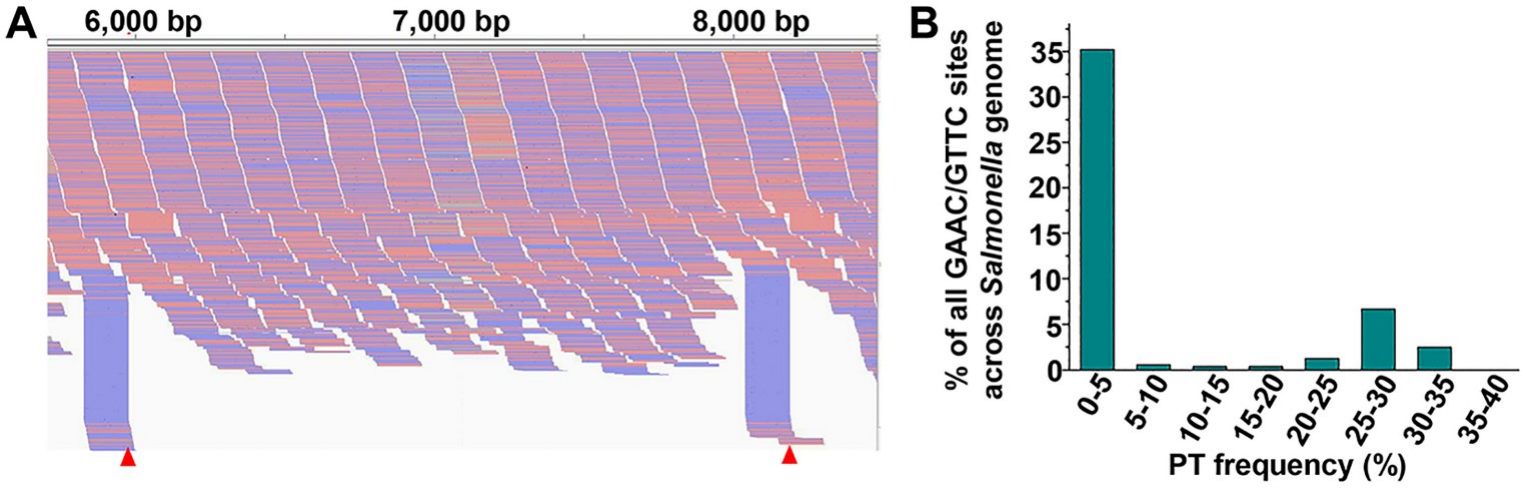
PT-IC-seq of *S*. *enterica serovar* Cerro 87 genome reveals low PT modification frequency globally. (A) The snapshot of genome browser representing partially modification site in bacteria genome. The top track shows counts of 5’ ends in the selected region. Blue segments represent reads mapped to minus strand and red segments represent reads mapped to plus strand. The red triangle marks two partial PT sites in *S*. *enterica serovar* Cerro 87 genome. (B) The ratio of the I_2_-specific-cleavaged reads to all the reads across the site and the PT ratio falling in the range of proportions. Statistics analysis of the PT modification frequency in the genomes of *S*. *enterica serovar* Cerro 87. All the PT modified sites in the genomes of *S*. *enterica serovar* Cerro 87 are also partially-PT modified, and about 35% of the PT modified sites with the PT ratio lower than 5%.

After confirming the heterogeneity feature of PT modification in *E*. *coli* B7A, we then attempt to explore whether the heterogeneity of PT modification is ubiquitous phenomena among other PT modified strains. The PT-IC-Seq method was then applied to the genomes of *S*. *enterica* serovar Cerro 87. By aligning the sequencing output reads to the *S*. *enterica* serovar Cerro 87 reference genome (GenBank accession No. NZ_CP008925.1), we found that in addition to these reads ended at each PT site, there were also a greater number of reads crossing over the same sites just as in *E*. *coli* B7A genome (Fig 6A). The existence of both the reads ended and crossing over at the same sites, suggests that the genomic PT modification in the *S*. *enterica* serovar Cerro 87 shows heterogeneity as well. After calculating the ratio of the iodine induced cleaved reads to all of the reads across or ended at this site, we found that all of the detected PT modified sites in *S*. *enterica* serovar Cerro 87 genome were also partially modified, and the percentage were also below 40% (S5 Table and Fig 6B). And about 35% of PT modified sites were modified with a PT modification percentage below 5%. The fact that PT modification in *S*. *enterica* serovar Cerro 87 also is characteristic of heterogeneity suggests that heterogeneity might be ubiquitous among PT modified strains. The quantitative mapping of PT sites in the *Salmonella* genome further supports that the PT-IC-seq method is a valuable tool to study PT-modified strains.

## Discussion

DNA and RNA modifications play critical roles in various biological processes, including R-M systems serving to protect bacteria against bacteriophages in prokaryotes, and as epigenetic marks for DNA replication, repair, recombination, chromatin organization and hypermutation in all organisms[23]. The best known DNA modifications in organisms are methylation of cytosine to produce 5-methylcytosine (m^5^C) and adenine to N6-methyladenine (m^6^A). The recently discovered PT modification of the DNA backbone in bacteria is an unusual DNA physiological modification, in that the nonbridging oxygen in the sugarphosphate backbone of DNA is replaced by sulfur. Benefiting from the latest developments of SMRT and ICDS sequencing methods, PT modified sites across the *E*. *coli* B7A genome have been mapped in recent studies [10]. The well characterization of the genomic landscape of PT modifications in bacteria have led to a greatly enhanced interest in the biology of PT. However, a quantitative map at single-base resolution of the PT modification percentage information is key to understand its biological function. Here, we developed PT-IC-seq and PT-IC-ddPCR to quantify PT modification sites in the *E*. *coli* B7A genome. As illustrated in Fig 2, PT-IC-seq combined iodine-induced cleavage at PT sites and high-throughput NGS was first used to quantitatively characterize the PT modification at the scale of whole genome. The feasibility of the PT-IC-seq technique was demonstrated in not only plasmid but also genomic DNA (Figs 2 and 3). Similar to m^6^A-RE-seq, an approach that uses DpnI to cleave methylated adenine sites in duplex DNA[13, 24], PT-IC-seq uses iodine reagent to cleave double-strand PT modified DNA. Iodine reagent sensitively and specifically cleaving PT-containing DNA have been demonstrated[10], and it only cleaves fully PT modified sites, thus PT-IC-seq strategy was limited to genomic mapping of bistranded PT modifications but not the single-stranded modifications. Although SMRT sequencing can distinguish modified PT from unmodified DNA and has been applied in genomic mapping of PT, the relatively high expense, low detection signals (the kinetic interpulse duration signals for PT modifications smaller than m^6^A signals) and the use of subjective thresholds[10] have prevented its wide applications in genomic mapping of PT. Comparing to ICDS sequencing, PT-IC-seq can quantitatively map PT modifications because the ratio of a specific GAAC or GTTC sites with sequence reads at the end versus internal represents the relative PT modified to unmodified ratio, which ICDS sequencing could not do. Furthermore, the PT modification frequency of specific target sites were absolutely quantified with the PT-IC-ddPCR to confirm the results of PT-IC-seq approach. Consistent with the PT-IC-seq results, both of them identified all eight sites have similar PT frequency. The low PT frequency sites based on PT-IC-seq results were determined to be low by PT-IC-ddPCR as well (S9 Table). PT-IC-ddPCR also showed good ability in quantifying the PT sites with low modification frequency. However, the relatively high expense and low throughput also have hindered their applications in genomic mapping of PT. Furthermore, the LC-MS/MS method developed previously, although sensitive enough to detect and accurately quantify the amount of the very low abundance of PT modifications, can only provide the overall ratio of the modification in total DNA. Therefore, PT-IC-seq method combining high throughput sequencing and bioinformatics analysis is a powerful strategy to quantitatively map PT modification sites in bacteria genome and to explore the PT biology.

We then applied this PT-IC-seq strategy to *E*. *coli* B7A and *S*. *enterica* serovar Cerro 87 genomic DNA and obtained genome-wide PT maps at single-nucleotide resolution that show PT heterogeneity in bacterial populations (Fig 3 and S5 Table). This PT heterogeneity phenomenon in bacterial populations is similar to recent observation of DNA methylation abundance in several distinct prokaryotes and even eukaryotes [12]. In *E*. *coli*, *Chromohalobacter salexigens*, *Geobacter metallireducens*, *Campylobacter jejuni* and *Helicobacter pylori*, the detected m^6^A or m^4^C methylations reveal distinct types of epigenetic heterogeneity[12]. In *Plasmodium falciparum* and *Penicillium chrysogenum*, most of the G(m^6^A)TC sites were partially methylated with a low percentage of methylation ratio (below 10%)[13]. Given the fact that solitary PT-modifying enzyme occur in more than half of all PT bacterial genome without an associated restriction enzyme component, PT heterogeneity in *E*. *coli* B7A genome suggests that PT modification may work as an marker other than R-M system. Heterogeneity is beneficial for organisms, because it enables some cells to survive in harsh environments. When sudden changes in chemical composition [25], local temperature [26] or other environment conditions occur, the heterogeneous population may contain some individuals that could cope with it, and thereby maintain the survival of the whole population. Hence, heterogeneity increases the overall population fitness when environmental shifts are unpredictable. As other organisms heterogeneity, PT heterogeneity may increase population-level phenotypic plasticity in response to changing environmental conditions or better preservation of certain functions of PT modification in the population to ultimately maintain the survival advantage of the population. These issues are yet to be revealed and more research is needed to answer these questions. Other than the roles of the PT heterogeneity in bacteria, the mechanism of heterogeneity for modification is also unclear. We propose two possible mechanisms for this PT heterogeneity phenomenon. One possibility is that, intracellular PT-modifying enzymes, which function as a large protein complex[5], could keep at a low activity level. The other possibility is that endogenous oxidants, metals or alkylating agents result in nonspecific desulfuration of PT modified DNA[27].

In summary, we developed PT-IC-seq and PT-IC-ddPCR approaches to quantify PT modification sites in bacteria genome. With these two methods, we clearly demonstrated that the widespread PT modification has heterogeneity in bacterial population which has important implications for the future study of PT modification.

## Materials and methods

### Materials and bacterial strains

The following materials were obtained from Sangon Biotech Co. Ltd. (Shanghai, China): Enantiomerically pure d(G_ps_A) and d(G_ps_T) in *R*_p_ and *S*_p_ configuration, the custom oligodeoxynucleotides duplex tag which marked the iodine nick in the ICDS, the primers and probes for PT-IC-ddPCR were listed in S6 Table. The TaqMan probes which were labeled with 6-carboxyfluorescein (FAM) at the 5’ end and black hole quencher (BHQ I) at the 3’ end. The plasmid pBluescript SK(+) was obtained from Life Technologies (Grand Island, NY, USA). *Salmonella enterica* serovar Cerro 87 was supplied by Professor Toshiyuki Murase (Tottori University, Japan), *E*. *coli* B7A was obtained from Dr Jaquelyn Fleckenstein (Departments of Medicine and Molecular Sciences, University of Tennessee Health Science Center) [2, 11]. Bacterial DNA Kit was purchased from TIANGEN (Cat. no. DP302-02 TIANGEN, Beijing, China). Plasmid Mini Kit and Cycle pure Kit were purchased from OMEGA (Omega Bio-tek, USA). The following kits and reagents were purchased from New England BioLabs (Ipswich, MA, USA): Antarctic Phosphatase, Quick Blunting Kit, Quick Ligation Kit, Klenow Fragment (3′-5′ exo^_^), dATP solution. Centrifugal filters (10 KD) were from Millipore (EMD Millipore, Billerica, MA, USA) and MicroSpin G-25 columns were from GE Healthcare (Buckinghamshire, UK). Iodine and nuclease P1 were from Sigma-Aldrich (St Louis, MO, USA). PCR tubes were from Molecular BioProducts (San Diego, CA, USA), Alkaline phosphatase were from Fermentas. 2×ddPCR Supermix and droplet generation oil were from Bio-Rad (Bio-Rad). All water was deionized and filtered using a MilliQ water purification system (EMD Millipore, Billerica, MA, USA).

### Quantitative determination of PT modification by LC-MS/MS

Phosphorothioate modifications in *E*. *coli* B7A were quantified by HPLC and LC-MS/MS (ACQUITY UPLC & SCIEX SelexION Triple Quad 5500 System). 20 μl of DNA was digested by nuclease P1 (Sigma-Aldrich, Cat. no. N8630) in 30 mM NH_4_OAc pH 5.3, 0.5 mM ZnCl_2_ in a 100 μl of total volume at 50 °C for 2 h. After the P1 hydrolyzation, 10 μl of 1 M Tris-HCl, pH 8.0 and 5 U of alkaline phosphatase (Fermentas, FASTAP) were added for the fully dephosphorylation at 37 °C for another 2 h. The enzymes were subsequently removed by ultrafiltration. Then the digested samples were dried and resuspended in 40 μl of deionized water.

The hydrolyzed mono nucleosides subsequently quantified by HPLC according to standard curves of pure A, T, C, and G nucleosides. Meanwhile, the PT-modified dinucleotides were quantified by LC-MS/MS according to standard curves of pure Rp configuration PT linked dinucleotides G_ps_A/G_ps_T(*R*p) and *S*p configuration PT linked dinucleotides G_ps_A/G_ps_T(*S*p) used as internal standards. Thus, the number of PT modified dinucleotides in a unit length of DNA can be calculated.

### ICDS sequencing and data analysis

Approximately 20 μg of purified genomic DNA was cleaved by I_2_ in a 100 μl reaction system which is consist of genomic DNA (20 μg), 50 mM Na_2_HPO_4_, 3 mM I_2_ at pH 9.0. Reaction system were incubated in 65 °C for 15 min and then slow cooled (0.1 °C/s) to 4 °C using a thermal cycler. Following iodine cleavage, the DNA segments were subjected to end repairment, 3’-deoxyadenylation and unique tag ligation at double-strand break sites according to instructions provided with the NEBNext DNA Library Prep Reagent Set for Illumina (New England BioLabs, Beverly, MA) which essentially described as follows: Terminal phosphates of the iodine cleaved DNA samples were removed with alkaline phosphatase (Fermentas, FASTAP (10 units)) at 37 °C for 60 min. Then the system was heated to 65 °C for 10 min for the inactivation of phosphatase, t and subsequently cooled to 4 °C slowly (0.1 °C s-1) to assure proper complementary re-annealing. Samples were cleaned up using cycle pure kit (OMEGA) and eluted with 30 μl MilliQ water. Break sites were blunt-ended using the Quick Blunting Kit at room temperature for 30 min. The enzyme was inactivated by heating the samples to 75 °C for 10 min and then slowly cooling them (0.1 °C s-1) to 4 °C. Samples were cleaned up using cycle pure kit and eluted with 30 μl MilliQ water. Next, blunt-ends were 3’-deoxyadenylated (that is, A-tailing) in reactions (100 μl) containing 1x NEB Buffer #2, 0.1 mM dATP, and Klenow fragment (3’-5’exo-) (15 units) at 37 °C for 30 min. The resulted system was treated as before and eluted with 30 μl MilliQ water. The enzyme was inactivated by heating the samples to 75 °C for 20 min and then slowly cooling them (0.1 °C s-1) to 4 °C. Finally, a custom 20-mer duplex tag-sequence (5’-FWD tag (5’-/Phos/TTTAACCGCGAATTCCAG /dideoxyC/-3’)/ 3’-REV tag (5’-GCTGGAATTCGC- GGTTAAAT-3’)) (3 μM) was ligated to 3’-deoxyadenylated ends using T4 ligase at 16 °C for 16 h. The enzyme was inactivated by heating and cooled to 4 °C as before, and the recovered sample with cycle pure kit was also resolved in 30 μl MilliQ water.

Then the quality of the samples was tested: the purity of the samples was measured using Nano Drop 2000; The concentration was measured using picogreen. Next, the DNA samples were sheared to fragments of optimal range (150–350 bp) for Illumina sequencing and followed by adaptor ligation. Unique tag marked and adaptor-ligated DNA segments were PCR amplified for 15 cycles and the segments which only have I_2_-cleaved ends for which the linked unique tag have been enriched. The prepared PE library was qualified and quantified by Qubit 3.0 Fluorometer (Life Technologies), agarose gel electrophoresis and Agilent 2100 bioanalyzer. The Library was sequenced on the Illumina HiSeq X Ten platform.

After Illumina sequencing completed, the reads which containing tag were selected and done adaptor and tag trimming and quality control as follows: 1) Clipping the adapter sequences; 2) Removing non-A, G, C, T bases of the 5 ' end; 3) Trimming low quality sequencing reads (base quality is less than Q20); 4) Removing reads with > 10% of“N”base calls; 5) Filtering small fragments less than 25 bp after clipping the adapter sequences and quality trimming. Then aligned to reference genomes by Burrows-Wheeler Aligner (BWA) and the position-wise coverage values were calculated using a custom python script. The GAAC/GTTC sites that only measured above 50 reads which ended at this site were regarded as PT modified sites. And 10 non GAAC/GTTC sites were randomly selected as control.

### PT-IC-seq and data analysis

Approximately 20 μg of genome DNA was cleaved by I_2_ as described above in ICDS methods. The cleaved DNA segments were further randomly sheared to fragments of optimal range (150–350 bp) for subsequent Illumina sequencing by sonication. After end-repaired by T4 DNA polymerase, 3’-adenylated by alkaline phosphatase, DNA segments were ligated Illumina adaptors and followed PCR amplified for 15 cycles. The prepared PE library was qualified and quantified by Qubit Fluorometer, agarose gel electrophoresis and Agilent 2100 bioanalyzer. The library was sequenced on the Illumina HiSeq X Ten platform.

After Illumina sequencing completed, all the reads were adaptor trimming and quality control. Then these reads were aligned to referred genomes by Burrows-Wheeler Aligner (BWA). The resulted alignment SAM files were converted to BAM files though Samtools. Visual inspection of the mapped data was performed by Integrated Genomics Viewer 2.3 software (IGV; Broad Institute, Cambridge, MA, USA). Meanwhile, the position-wise reads number obtained from fragment terminals and across the same site were calculated separately using a custom python script. The PT ratio of each GAAC/GTTC sites were calculated by the number of reads ended at this site divided by all of the number of reads ended and crossed the same sites. To overcome the false-ended reads generated by random shearing, ten non GAAC/GTTC sites were randomly selected as control sites. The average of the PT ratios of the ten non GAAC/GTTC sites were calculated and treated as threshold. The PT ratio of each modified sites throughout of the genome were all calculated and only those sites which’s PT ratio passing the threshold are kept for further analysis.

### PT-IC-ddPCR quantitative assay

The DNA sample to be analyzed was divided into two equal parts, one part was iodine cleaved as described above and the other part of DNA treated with the same iodine cleavage reaction solution but without iodine. The treated DNA samples were qualified using ddPCR. The ddPCR reaction was performed using a QX200^™^ droplet digital PCR system (Bio-Rad). The dd-PCR assays were carried out with10 μl of 2×ddPCR Supermix (Bio-Rad), 1 μl of 10 μM forward primer, 1 μl of 10 μM reverse primer, 0.5 μl of 10 μM probes and 1 μl of DNA template, complemented with DNase free water for the final volume of 20 μl. The 20 μl reaction mixture was loaded into a 8-well cartridges of the DG8^™^ cartridge (Bio-Rad) together with 70 μl of droplet generation oil (Bio-Rad). A QX200^™^ Droplet Generator (Bio-Rad) was used to dispense the 20 μl ddPCR reaction mixtures into droplets. Then the water-in-oil droplet emulsions (40 μl) were further transferred to a 96-well PCR reaction plate (Eppendorf) and amplified in a T100^™^ Thermal Cycler (Bio-Rad). The thermal parameters were as follows: 95 °C for 5 min, followed by 40 cycles of 30 s at 95 °C and 1 min at 55 °C; followed by 10 min at 98 °C for 10 min and a hold at 4 °C. After PCR amplification, the 96-well plates were transferred to the QX200^™^ droplet reader (Bio-Rad Laboratories). Data acquisition and analysis was performed using the Quanta Soft (Bio-Rad Laboratories) droplet reader software. Positive droplets distinguished from negative droplets by the threshold determined according to the Quanta Soft software (S2 Fig). The number of copies per μl of PCR reaction mixture were estimated according to the number of positive droplets and the total number of partitions. The precision of the ddPCR depends on the number of partitions generated. Wells with less than 10,000 accepted droplets were excluded from analysis. The original number of copies in the sample were obtained by ddPCR software and multiplied by 20 (20 μl ddPCR reaction system). The concentration values obtained from the ddPCR was converted as log10 (number of copies/μl+1) to obtain the standard curve. In each run, the reaction of the iodine cleavage DNA and the non-iodine treated DNA along with a no template control (NTC) were performed. All reactions were performed with triplicates, and each experiment was repeated three times on three different days.

## Supporting information

S1 Fig Iodine cleavage of PT DNA. (A-C) Electrophoresis pattern of the iodine-cleaved genomic or plasmid DNA (lane 2); iodine repleaded with ethanol as a negative control (lane 1); M, DNA ladder. (A) Iodine-cleaved genomic DNA from *E*. *coli* B7A for ICDS and PT-IC-seq; (B) Iodine-cleaved pBlueScript SK(+) plasmid DNA extract from *S*. *enterica* serovar Cerro 87 for PT-IC-seq; (C) Iodine-cleaved genomic DNA from *S*. *enterica* serovar Cerro 87 for PT-IC-seq. (D) MALDI-TOF analysis of ethanol and iodine-ethanol cleavage of 48-mer PT-containing oligodeoxynucleotides. Oligonucleotides with the same sequence as in previous cleavage studies (10) were used. In the ethanol treated control group, the *m*/*z* of the 48-mer PT containing oligonucleotides was tested as 29480.4 [M+H]^+^, 14826.1 [M+H]^2+^ and 7425.4 [M+H]^4+^. However, in the iodine treated group, no signal of 29480.4, 14826.1 and 7425.4 could be found.
(TIF)

S2 Fig PT-IC-ddPCR analysis for the quantification of PT modification frequency at 1818096 locus in *E*. *coli* B7A genome. (A/C) The ddPCR detection of the non-iodine treated and the iodine cleavage DNA. (B/D) The ddPCR statistical analysis of the fluorescence signals of the non-iodine treated and the iodine cleavage DNA.
(TIF)

S1 Table ICDS sequencing analysis of phosphorothioate modifications in the genome of *Escherichia coli* B7A. (XLSX)

S2 Table Statistics analysis of PT modification at representative sites with gradient increase of sequencing depth. Each specific site displayed here is to exemplify the different detected PT modification situations with the gradient sequencing depth.
(PDF)

S3 Table The ended reads number at randomly selected 10 non GAAC/GTTC sites. The ended reads number at randomly selected 10 non GAAC/GTTC sites maintained almost zero and didn’t increase with the sequencing depth increased.
(PDF)

S4 Table PT ratio of each PT sites across the pBlueScript SK(+) plasmid extract from *S*. *enterica* serovar Cerro 87. (XLSX)

S5 Table PT ratio of each PT sites across bacteria genome. (XLSX)

S6 Table Overlapped PT modified sites with different modification frequency of PT-IC-seq and ICDS approach with 200 × coverage. (PDF)

S7 Table Analysis of consensus sequences in *E*. *coli* B7A. (PDF)

S8 Table DNA primers and probes used in PT-IC-ddPCR. (PDF)

S9 Table 8 loci of the genome of *E*. *coli* B7A quantified PT modification frequency. Eight G_ps_AAC/G_ps_TTC loci on the genome of *E*. *coli* B7A were randomly selected to test the phosphorothioate modification frequency by PT-IC-ddPCR and PT-IC-qPCR. And compared with the modification frequency obtained by PT-IC-seq.
(PDF)
