# Supplementary figures and images for: Quantitative mapping of DNA phosphorothioatome reveals phosphorothioate heterogeneity of low modification frequency
Source: PLoS Genet. 2019 Apr 1;15(4):e1008026. doi: 10.1371/journal.pgen.1008026 (PMC6459556; doi:10.1371/journal.pgen.1008026)

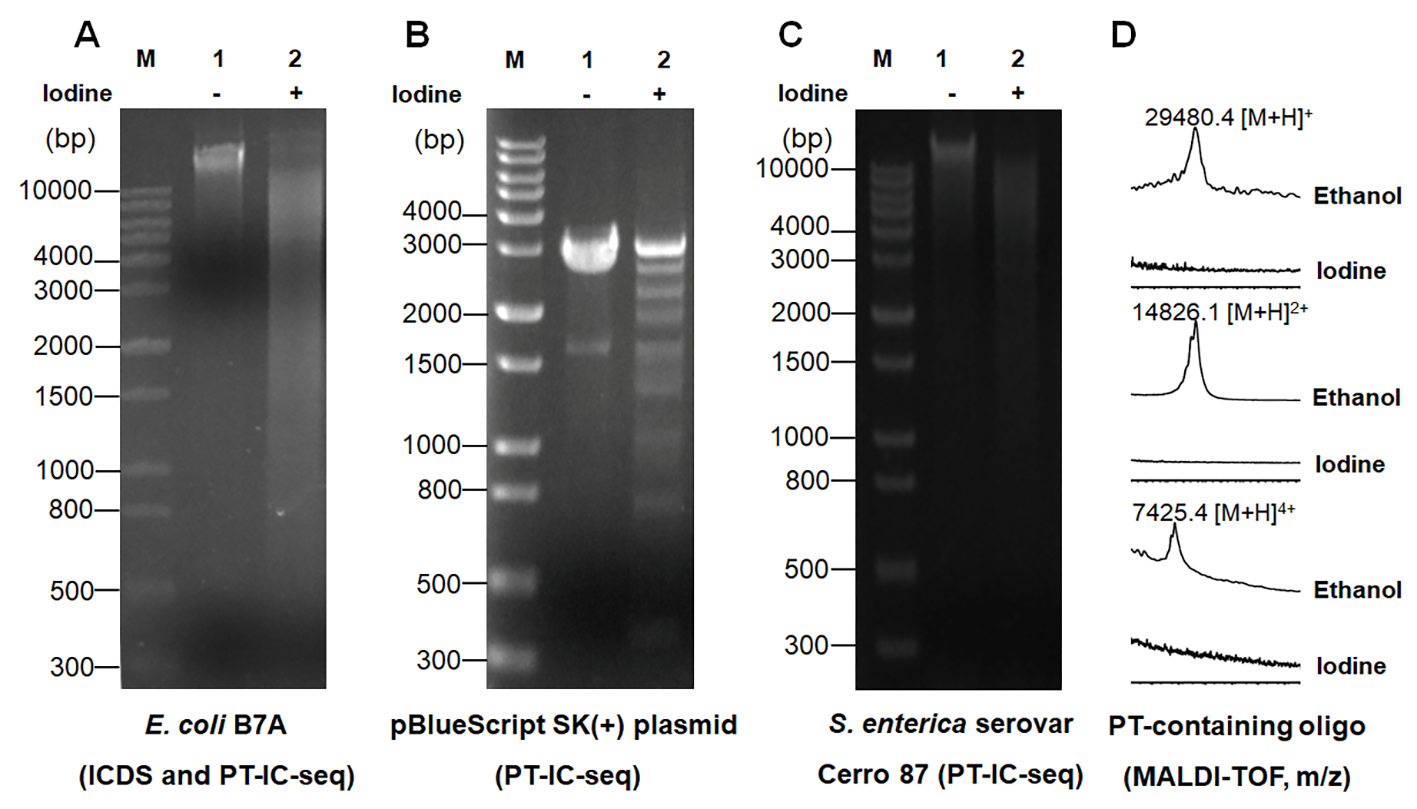

Supplement: S1 Fig — (A-C) Electrophoresis pattern of the iodine-cleaved genomic or plasmid DNA (lane 2); iodine repleaded with ethanol as a negative control (lane 1); M, DNA ladder. (A) Iodine-cleaved genomic DNA from E. coli B7A for ICDS and PT-IC-seq; (B) Iodine-cleaved pBlueScript SK(+) plasmid DNA extract from S. enterica serovar Cerro 87 for PT-IC-seq; (C) Iodine-cleaved genomic DNA from S. enterica serovar Cerro 87 for PT-IC-seq. (D) MALDI-TOF analysis of ethanol and iodine-ethanol cleavage of 48-mer PT-containing oligodeoxynucleotides. Oligonucleotides with the same sequence as in previous cleavage studies (10) were used. In the ethanol treated control group, the m/z of the 48-mer PT containing oligonucleotides was tested as 29480.4 [M+H]+, 14826.1 [M+H]2+ and 7425.4 [M+H]4+. However, in the iodine treated group, no signal of 29480.4, 14826.1 and 7425.4 could be found. (TIF) [file pgen.1008026.s001.tif]

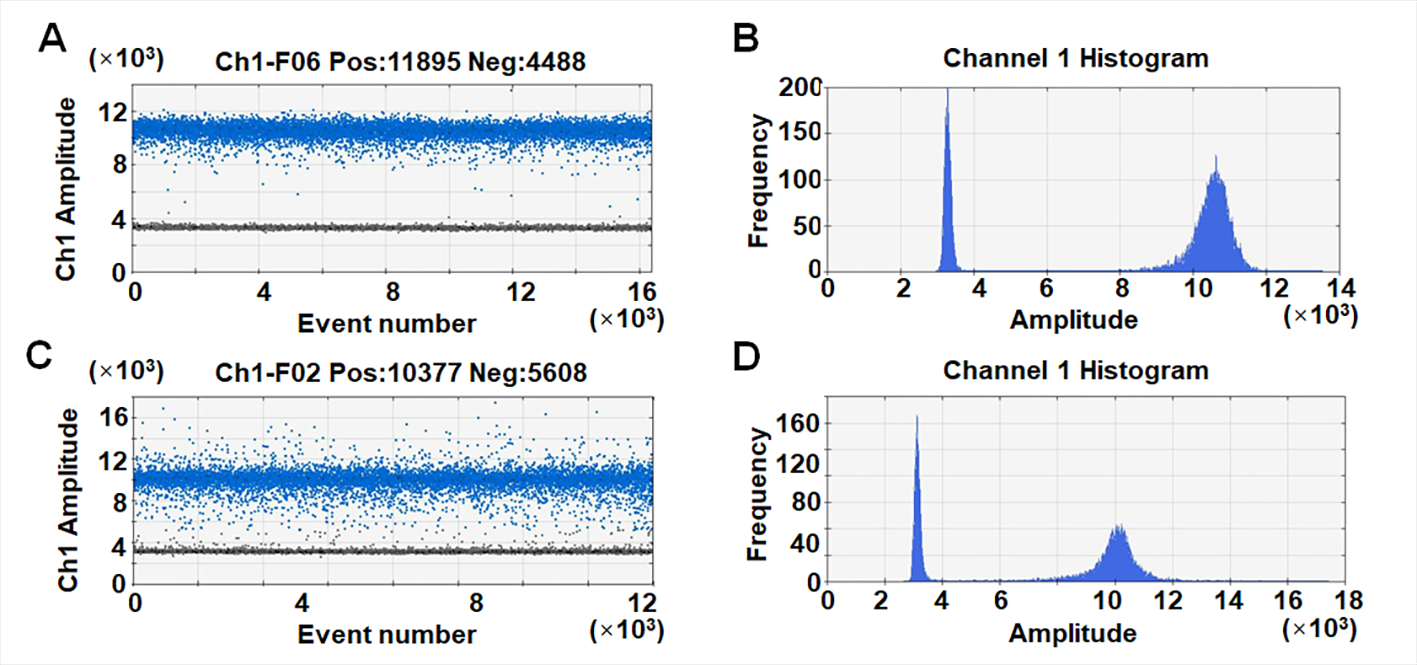

Supplement: S2 Fig — (A/C) The ddPCR detection of the non-iodine treated and the iodine cleavage DNA. (B/D) The ddPCR statistical analysis of the fluorescence signals of the non-iodine treated and the iodine cleavage DNA. (TIF) [file pgen.1008026.s002.tif]
