# Supplementary material for: Quantitative mapping of DNA phosphorothioatome reveals phosphorothioate heterogeneity of low modification frequency
Source: PLoS Genet. 2019 Apr 1;15(4):e1008026. doi: 10.1371/journal.pgen.1008026 (PMC6459556; doi:10.1371/journal.pgen.1008026)
Supplement: S2 Table — Each specific site displayed here is to exemplify the different detected PT modification situations with the gradient sequencing depth. (PDF) [file pgen.1008026.s004.pdf]

S2 Table. Statistics analysis of PT modification at representative sites with gradient increase of sequencing depth. Each specific site displayed here is to exemplify the different detected PT modification situations with the gradient sequencing depth.

Position	Pattern	200 ×	400 ×	600 ×	800 ×	1000 ×
		Depth of reads ended at specific site				
33670	GTTC	63	121	174	226	282
3865	GAAC	47	67	105	150	175
19366	GAAC	19	32	59	79	104
5390	GTTC	11	31	41	61	90
1138	GTTC	14	25	34	38	53
301	GTTC	8	15	18	23	39
614	GTTC	0	0	0	0	0
