# Supplementary material for: Quantitative mapping of DNA phosphorothioatome reveals phosphorothioate heterogeneity of low modification frequency
Source: PLoS Genet. 2019 Apr 1;15(4):e1008026. doi: 10.1371/journal.pgen.1008026 (PMC6459556; doi:10.1371/journal.pgen.1008026)
Supplement: S3 Table — The ended reads number at randomly selected 10 non GAAC/GTTC sites maintained almost zero and didn’t increase with the sequencing depth increased. (PDF) [file pgen.1008026.s005.pdf]

**S3 Table. The ended reads number at randomly selected 10 non GAAC/GTTC sites.** The ended reads number at randomly selected 10 non GAAC/GTTC sites maintained almost zero and didn't increase with the sequencing depth increased.

| Position | Pattern | 200 × | 400 × | 600 × | 800 × | 1000 × |
|---|---|---|---|---|---|---|
| | | Depth of reads ended at specific site | | | | |
| 368 | CCGA | 0 | 0 | 0 | 0 | 2 |
| 2042 | TTGA | 0 | 0 | 0 | 0 | 0 |
| 2357 | ACGG | 0 | 0 | 0 | 0 | 0 |
| 3144 | AGGC | 0 | 0 | 0 | 0 | 0 |
| 5147 | GCGT | 0 | 0 | 0 | 0 | 0 |
| 8661 | CAAC | 0 | 2 | 2 | 3 | 5 |
| 13139 | TTTC | 2 | 3 | 2 | 0 | 1 |
| 21699 | ACCG | 0 | 0 | 0 | 0 | 0 |
| 25667 | CAGT | 0 | 0 | 0 | 0 | 0 |
| 30950 | ATAC | 0 | 0 | 0 | 0 | 0 |
