# Supplementary material for: Quantitative mapping of DNA phosphorothioatome reveals phosphorothioate heterogeneity of low modification frequency
Source: PLoS Genet. 2019 Apr 1;15(4):e1008026. doi: 10.1371/journal.pgen.1008026 (PMC6459556; doi:10.1371/journal.pgen.1008026)
Supplement: S6 Table — (PDF) [file pgen.1008026.s008.pdf]

S6 Table. Overlapped PT modified sites with different modification frequency of PT-IC-seq and ICDS approach with 200 × coverage.

PT ratio	Sites number	Overlaped with 200 ×	Percentage
⩾30	360	353	98.06
⩾25	1608	1596	99.25
⩾20	2718	2699	99.30
⩾15	3714	3673	98.90
⩾10	5379	5261	97.81
⩾6	8725	7750	88.83
