# Supplementary material for: Quantitative mapping of DNA phosphorothioatome reveals phosphorothioate heterogeneity of low modification frequency
Source: PLoS Genet. 2019 Apr 1;15(4):e1008026. doi: 10.1371/journal.pgen.1008026 (PMC6459556; doi:10.1371/journal.pgen.1008026)
Supplement: S7 Table — (PDF) [file pgen.1008026.s009.pdf]

S7 Table. Analysis of consensus sequences in *E. coli* B7A

GAAC/GTTC		Downstream base			
		A	C	G	T
Upstream base	A	2.78% (10/360)	4.17% (15/360)	23.61% (85/360)	5.56% (20/360)
	C	3.61% (13/360)	4.17% (15/360)	11.67% (42/360)	3.89% (14/360)
	G	3.89% (14/360)	2.78% (10/360)	21.67% (78/360)	5% (18/360)
	T	0.28% (1/360)	1.39% (5/360)	4,72% (17/360)	0.83% (3/360)
