# Supplementary material for: Quantitative mapping of DNA phosphorothioatome reveals phosphorothioate heterogeneity of low modification frequency
Source: PLoS Genet. 2019 Apr 1;15(4):e1008026. doi: 10.1371/journal.pgen.1008026 (PMC6459556; doi:10.1371/journal.pgen.1008026)
Supplement: S8 Table — (PDF) [file pgen.1008026.s010.pdf]

S8 Table. DNA primers and probes used in PT-IC-ddPCR

Site	Genome position	Primer/ probes	Primer sequence (5`-3`)	Product size (bp)
G1	21687	G1-F	GACCCAGTATGTCAACCCAACC	142
		G1-R	GGAATTCTGGACCGCTGTCG	
		G1P	ACAAACCACCACCGATAATGTTGCCGATC	
G2	607710	G2-F	GATGGCTTCGTTAAGTGTTAGTCC	109
		G2-R	GCTGACTCTGACATTATGGTATCG	
		G2P	CCTGTTCTCACCGCATGGTCAACGCC	
G3	1201555	G3-F	TGGCAATTCATATATTGGCCTGAC	134
		G3-R	TGAAGTGCTACTCCCCTTTGTG	
		G3P	CTGGAAAACCCGCCTCACGCATCATAAATG	
G4	1818096	G4-F	ACGCAATTCACGTACTGACATG	124
		G4-R	AAAAGCCAAACTTTTCGAATTAATGAC	
		G4P	ATCAACATCGTCAAGCGTCATGCCGG	
G5	3592199	G5-F	AAGACTCAACTGTTTAATGAGATGTGG	98
		G5-R	TGAAACGGGTCATGCTCTAATCC	
		G5P	CAGCCTTCATTGCGGGAGTTAATCCAGGAAC	
G6	4585867	G6-F	TGTTGATGGTAGTAACGACTTTGC	140
		G6-R	GTATCCCATGCGGGTATCCTG	
		G6P	CTTTTCTGCCCCTGGATGGAGCAAACCC	
G7	3026955	G7-F	ATCAGAGAGAGAAGACCGAAACC	119
		G7-R	CCACGAGTACACCTCTCCTTAG	
		G7P	TCATCGTGAATCCATTAGACTTAGAAAATATCGGGTC	
G8	4120753	G8-F	TAAAGATGGATGGGCAGATCGG	144
		G8-R	GGATCACGAAAAGTATCTCTGGAC	
		G8P	CGCCTCGTTCGCCTTTGCCGCC	
