# Supplementary material for: Quantitative mapping of DNA phosphorothioatome reveals phosphorothioate heterogeneity of low modification frequency
Source: PLoS Genet. 2019 Apr 1;15(4):e1008026. doi: 10.1371/journal.pgen.1008026 (PMC6459556; doi:10.1371/journal.pgen.1008026)
Supplement: S9 Table — Eight GpsAAC/GpsTTC loci on the genome of E. coli B7A were randomly selected to test the phosphorothioate modification frequency by PT-IC-ddPCR and PT-IC-qPCR. And compared with the modification frequency obtained by PT-IC-seq. (PDF) [file pgen.1008026.s011.pdf]

S9 Table. 8 loci of the genome of *E. coli* B7A quantified PT modification frequency. Eight $G_{ps}AAC/G_{ps}TTC$ loci on the genome of *E. coli* B7A were randomly selected to test the phosphorothioate modification frequency by PT-IC-ddPCR and PT-IC-qPCR. And compared with the modification frequency obtained by PT-IC-seq.

Genome position	Type	Function	Subsystems	PT frequency (%)	
				PT-IC-ddPCR	PT-IC-seq
21687	Gene	formate transporter	Inorganic ion transport and metabolism	30.35	25.87
607710	Gene	benzoate transporter	Secondary metabolites biosynthesis, transport, and catabolism	46.43	28.44
1201555	Gene	hypothetical protein	Cell envelope biogenesis, outer membrane	49.69	32.63
1818096	Gene	Gp41	Glycoprotein	8.78	5.19
3592199	Gene	EnvR	DNA-binding transcriptional regulator	47.55	31.01
4585867	Gen	putative protease	Posttranslational modification, protein turnover, chaperones	39.00	29.29
3026955	Promoter	Rep	ATP-dependent DNA helicase	12.1	5.45
4120753	Gene	hypothetical protein	Function unknown	15.49	9.55
